# Barriers and Facilitators Influencing Participation in Continuing Nursing Education Programs Among Nursing Officers at a Tertiary Care Center: A Descriptive Cross-Sectional Study

**DOI:** 10.7759/cureus.112709

**Published:** 2026-07-15

**Authors:** Suman Choudhary, Anita Rani, Barun Kumar, Vandana Vandana, Jino Jacob, Arun Ravi, Pankaj Rana, Prakash Mahala

**Affiliations:** 1 Continuing Nursing Education (CNE) Cell, All India Institute of Medical Sciences, Rishikesh, IND; 2 Nursing Services, All India Institute of Medical Sciences, Rishikesh, IND; 3 Cardiology, All India Institute of Medical Sciences, Rishikesh, IND; 4 Dialysis Unit, All India Institute of Medical Sciences, Rishikesh, IND; 5 Emergency Medicine, All India Institute of Medical Sciences, Rishikesh, IND

**Keywords:** barriers, continuing nursing education (cne), facilitators, nursing, nursing officer

## Abstract

Introduction: Continuing Nursing Education (CNE) is an essential aspect of lifelong learning that helps nurses improve their knowledge, clinical skills, and professional competence for quality patient care. Active participation in CNE programs supports evidence-based nursing practice and enhances healthcare outcomes. However, the involvement of nursing officers in continuing education activities is often influenced by several individual, workplace, and organizational factors, which may either encourage or limit their participation.

Methods: A descriptive cross-sectional study using the convenience sampling technique was conducted at All India Institute of Medical Sciences, Rishikesh, across various clinical departments to assess the barriers and facilitators influencing participation in CNE programs among nursing officers. A total of 383 nursing officers participated in the study.

Results: The data analysis revealed that the majority of nursing officers demonstrated high awareness regarding CNE programs (334; 87.2%). Heavy workload and staff shortage during duty hours were identified as the major barriers influencing participation in CNE activities (mean ± standard deviation (SD): 4.03 ± 0.97), followed by lack of time due to patient care responsibilities (3.79 ± 1.03) and fatigue after long shifts (3.32 ± 1.21). The most important facilitators encouraging participation were clinically relevant and updated topics (4.13 ± 0.84), flexible scheduling (4.04 ± 0.82), peer encouragement (4.04 ± 0.88), and supportive learning environments (4.03 ± 0.92). The analysis also showed no statistically significant association between demographic variables and awareness regarding CNE participation among nursing officers (p > 0.05).

Conclusion: The study's findings indicate that workload, staff shortage, and time constraints were the major barriers to participation in CNE programs among nursing officers. Flexible scheduling, peer support, and interactive learning methods were identified as important facilitators that encouraged participation in CNE activities.

## Introduction

Continuing Nursing Education (CNE) is an important component of professional development that enables nurses to update their knowledge, improve clinical competence, and maintain high standards of patient care. Continuous advancements in healthcare technology, disease management, and evidence-based practices have increased the need for ongoing learning among nursing professionals [1]. Participation in CNE programs enhances clinical decision-making, professional confidence, critical thinking abilities, and quality of healthcare delivery [2].

Despite the recognized importance of continuing education, participation in CNE activities among nursing officers is often affected by several personal, professional, and organizational factors. Studies have reported that heavy workload, staff shortages, insufficient time during duty hours, fatigue after long shifts, and inconvenient scheduling of educational sessions are major barriers to participation in continuing education programs [3,4]. Limited administrative support and inadequate communication regarding educational activities have also been identified as factors reducing nurses' participation in professional development programs [5].

On the other hand, several facilitators have been shown to improve participation in CNE programs. Flexible scheduling, supportive leadership, peer encouragement, duty adjustment, and availability of online learning opportunities positively influence nurses’ involvement in continuing education activities [6]. Interactive teaching strategies such as case-based discussions, simulation training, and skill-oriented sessions have also been reported to increase interest and participation among nursing officers [7].

Evidence from recent studies indicates that strengthening CNE among nurses contributes to improved patient safety, better clinical outcomes, and enhanced professional competency [8]. A recent scoping review emphasized that organizational encouragement and accessible educational opportunities are essential for sustaining nurses’ motivation toward lifelong learning [9].

Although several international studies have explored factors influencing participation in continuing education, limited evidence is available regarding the barriers and facilitators affecting participation in CNE programs among nursing officers in India. Therefore, the present study was undertaken to identify the key barriers and facilitators influencing participation in CNE programs among nursing officers at the All India Institute of Medical Sciences (AIIMS), Rishikesh, assess their level of awareness and attitudes toward CNE, and determine the association between selected demographic variables and participation in and awareness of CNE.

## Materials and methods

A descriptive cross-sectional study was conducted to assess the barriers and facilitators influencing participation in CNE programs among nursing officers at AIIMS, Rishikesh. The required sample size was calculated using the single population proportion formula, assuming a 95% confidence level (Z = 1.96), a 5% margin of error (d = 0.05), and an anticipated proportion (p = 0.50), which yielded a minimum required sample size of approximately 383 participants. A total of 383 nursing officers were included in the study. Participants were selected using convenience sampling. The study population comprised nursing officers working across various clinical departments at AIIMS, Rishikesh. Nursing officers who were available during the data collection period and willing to participate in the study were included.

Data collection

Data were collected from February 21 to April 30, 2026, using a self-structured, self-administered questionnaire. The questionnaire was developed following an extensive review of the literature. Content validity was established through expert review by seven nursing and healthcare professionals with expertise in the relevant field, yielding a content validity index (CVI) of 0.72. A pilot study was conducted with 25 nursing officers to assess the instrument's reliability. Internal consistency was evaluated using Cronbach's alpha coefficient, which was 0.75, indicating acceptable reliability. Data obtained during the pilot study were excluded from the final analysis.

The questionnaire consisted of four sections: Section A: Demographic variables; Section B: Awareness and attitude toward CNE (10 items); Section C: Perceived barriers to participation in CNE (10 items); and Section D: Perceived facilitators for participation in CNE (10 items). The complete questionnaire used for data collection is provided in the appendix.

Scoring of the questionnaire

Responses to Sections B, C, and D were recorded using a five-point Likert scale, where 1 = strongly disagree, 2 = disagree, 3 = neutral, 4 = agree, and 5 = strongly agree. The total awareness score ranged from 10 to 50 and was categorized as follows: low awareness: 10-23; moderate awareness: 24-37; high awareness: 38-50. Higher scores indicated greater awareness and more positive attitudes toward CNE. For barrier items, higher scores indicated stronger perceived barriers to participation in CNE programs, whereas for facilitator items, higher scores indicated stronger facilitating factors encouraging participation.

Ethical considerations

Ethical approval was obtained from the Institutional Ethics Committee (AIIMS/IEC/26/65/13/02/2026) prior to data collection. Participants were informed about the study's purpose, and written informed consent was obtained prior to data collection.

Statistical analysis

The collected data were entered into Microsoft Excel (Microsoft Corp., Redmond, WA, USA) and analyzed using IBM SPSS Statistics version 23.0 (IBM Corp., Armonk, NY, USA). Descriptive statistics were used to summarize the data. Categorical variables such as age, gender, professional experience, and participation in CNE programs were presented as frequency (n) and percentage (%). Responses related to barriers, facilitators, and awareness/attitudes toward CNE were summarized using mean and standard deviation (mean ± SD). Inferential statistics were performed using the Chi-squared test to determine the association between selected demographic variables and awareness regarding CNE. A p-value of less than 0.05 was considered statistically significant.

## Results

Table 1 shows that 383 nursing officers participated in the study. The majority of participants were aged 25-35 years (330; 86.2%) and were female (228; 59.5%). Most participants had 1-5 years of professional experience (176; 46.0%). A high proportion of nursing officers (364; 95.0%) reported participation in CNE programs, indicating strong engagement in professional development activities.

**Table 1:** Frequency and percentage distribution of the respondents (n = 383) CNE: Continuing Nursing Education

Parameters	Category	Frequency	Percentage
Age	25-35 years	330	86.2%
	36-45 years	38	9.9%
	Below 25 years	15	3.9%
Gender	Female	228	59.5%
	Male	155	40.5%
Professional experience	1-5 years	176	46.0%
	6-10 years	140	36.6%
	More than 10 years	46	12.0%
	Less than 1 year	21	5.5%
Participation in CNE	Yes	364	95.0%
	No	19	5.0%

As shown in Table 2, heavy workload and staff shortage during duty hours (mean ± SD: 4.03 ± 0.97) were the major barriers influencing participation in CNE programs among nursing officers. Lack of time due to patient care responsibilities (3.79 ± 1.03) and fatigue or burnout after long shifts (3.32 ± 1.21) were also identified as important barriers. Inconvenient scheduling of CNE sessions (3.22 ± 1.08) moderately affected participation. Other barriers such as inadequate information regarding sessions (2.77 ± 1.12), personal commitments (2.67 ± 1.11), inconvenient venue or location (2.57 ± 1.10), lack of encouragement from supervisors (2.40 ± 1.07), repetitive sessions (2.28 ± 0.98), and irrelevance of topics to the current work area (2.16 ± 1.03) showed comparatively lower mean scores. Overall, workload, staff shortage, and time-related factors emerged as the primary barriers affecting participation in CNE programs among nursing officers.

**Table 2:** Perceived barriers to participation in CNE (n = 383) Responses were measured using a five-point Likert scale: 1 = strongly disagree, 2 = disagree, 3 = neutral, 4 = agree, and 5 = strongly agree. Higher mean scores indicate greater perceived barriers to participation in CNE programs. CNE: Continuing Nursing Education

Barriers to participation in CNE	Mean score	Std. deviation
Heavy workload and staff shortage during duty hours	4.03	0.970
Lack of time due to patient care responsibilities	3.79	1.034
Fatigue or burnout after long shifts	3.32	1.219
CNE sessions are scheduled at inconvenient times	3.22	1.084
Inadequate information or short notice about sessions	2.77	1.122
Personal commitments sometimes prevent me from attending	2.67	1.117
The venue/space or location of CNE is not convenient	2.57	1.107
Not received enough encouragement from supervisors	2.40	1.073
Sessions are repetitive or not interesting	2.28	0.986
CNE topics are not related to my current work area	2.16	1.032

The findings in Table 3 indicate that nursing officers considered clinically relevant and updated topics as the strongest motivating factor for participation in CNE programs (mean ± SD: 4.13 ± 0.84). Factors such as flexible scheduling (4.04 ± 0.82), encouragement from colleagues (4.04 ± 0.88), and a supportive learning environment with adequate facilities (4.03 ± 0.92) also positively influenced participation. Recognition for attending CNE programs, practical teaching approaches including simulations and case discussions, and proper duty adjustment showed similarly high mean scores (4.01 ± 0.89, 4.01 ± 0.86, and 4.01 ± 0.89, respectively), reflecting their importance in motivating nursing officers. Additionally, availability of learning materials (3.87 ± 0.95), online or recorded sessions (3.79 ± 1.11), and support from seniors (3.79 ± 0.94) were identified as helpful factors encouraging participation. The findings suggest that supportive work environments, relevant educational sessions, and convenient learning arrangements positively influenced participation in CNE programs among nursing officers.

**Table 3:** Perceived facilitators to participation in CNE (n = 383) Responses were measured using a five-point Likert scale: 1 = strongly disagree, 2 = disagree, 3 = neutral, 4 = agree, and 5 = strongly agree. Higher mean scores indicate stronger facilitators encouraging participation in CNE programs. CNE: Continuing Nursing Education

Facilitators to attending CNE	Mean score	Std. deviation
Topics relevant to clinical practice and current updates	4.13	0.849
Flexible or well-planned timing would increase my participation	4.04	0.827
Peer encouragement and team participation	4.04	0.888
Comfortable learning environment and facilities	4.03	0.929
Recognition or appreciation for attending CNEs	4.01	0.899
Interactive and practical learning (case studies, simulations)	4.01	0.862
Duty adjustment or shift planning helps me participate	4.01	0.894
Provision of handouts or learning materials	3.87	0.957
Online or recorded sessions for those on duty/off shift	3.79	1.117
Support from seniors inspires me to attend CNE	3.79	0.944

Table 4 indicates that nursing officers had a positive awareness and attitude toward CNE programs. The highest mean score was observed for the statement that CNE programs are important for the professional development of nurses (mean ± SD: 4.38 ± 1.03), followed by the perception that attending CNE programs improves the quality of patient care (4.35 ± 1.00). Participants also agreed that CNE programs motivate them to update their clinical knowledge (4.23 ± 1.03) and that the topics covered in CNE sessions are relevant to their clinical practice (4.22 ± 1.03). Nursing officers expressed confidence in applying knowledge gained from CNE sessions in daily work (4.21 ± 1.00) and reported awareness regarding CNE programs conducted in the hospital (4.13 ± 1.05). Positive responses were also observed regarding recommending regular CNE attendance to colleagues (4.11 ± 1.01), the effectiveness of teaching methods used in CNE sessions (4.09 ± 1.03), and the need for mandatory participation in CNE for career advancement (4.06 ± 1.05). The statement regarding encouragement from hospital management also showed a favorable response (4.01 ± 1.07). The findings indicate that nursing officers possessed good awareness and favorable attitudes toward participation in CNE programs.

**Table 4:** Awareness and attitude toward CNE (n = 383) Responses were measured using a five-point Likert scale: 1 = strongly disagree, 2 = disagree, 3 = neutral, 4 = agree, and 5 = strongly agree. Higher mean scores indicate greater awareness and more positive attitudes toward Continuing Nursing Education (CNE) programs.

Awareness and attitude toward CNE	Mean score	Std. deviation
CNE programs are important for professional development of nurses	4.38	1.036
Attending CNE programs improves the quality of patient care	4.35	1.002
Attending CNE programs motivates me to update my clinical knowledge	4.23	1.033
The topics covered in CNE sessions are relevant to my clinical practice	4.22	1.037
I feel confident applying knowledge gained from CNE sessions in my daily work	4.21	1.009
I am aware of the CNE programs conducted in my hospital	4.13	1.054
I would recommend regular CNE attendance to my colleagues	4.11	1.014
The methods used in CNE sessions are effective for learning	4.09	1.033
CNE participation should be mandatory for career advancement or promotion	4.06	1.056
The hospital management encourages participation in CNE sessions	4.01	1.074

The data presented in Table 5 show that there was no statistically significant association between demographic variables and awareness regarding CNE among nursing officers, as all p-values were greater than 0.05. Age showed a Chi-squared value of 0.613 (df = 4, Cramer's V = 0.028, p = 0.962), gender showed a Chi-squared value of 0.390 (df = 2, Cramer's V = 0.032, p = 0.823), and years of professional experience showed a Chi-squared value of 5.027 (df = 6, Cramer's V = 0.081, p = 0.540). These findings indicate that awareness regarding CNE was similar across different age groups, genders, and levels of professional experience among nursing officers. Furthermore, the effect sizes were negligible, suggesting a very weak relationship between the selected demographic variables and awareness regarding CNE.

**Table 5:** Association between demographic variables and awareness regarding CNE χ² = Pearson Chi-squared test statistic; df = degrees of freedom; Cramer's V = effect size measure for the strength of association between variables. A p-value < 0.05 was considered statistically significant. CNE: Continuing Nursing Education

Personal variables	Chi square (χ²)	df	Cramer’s V	p-value
Age (in years)	0.61	4	0.028	0.962
Gender	0.39	2	0.032	0.823
Years of professional experience	5.027	6	0.081	0.540

**Figure 1:**
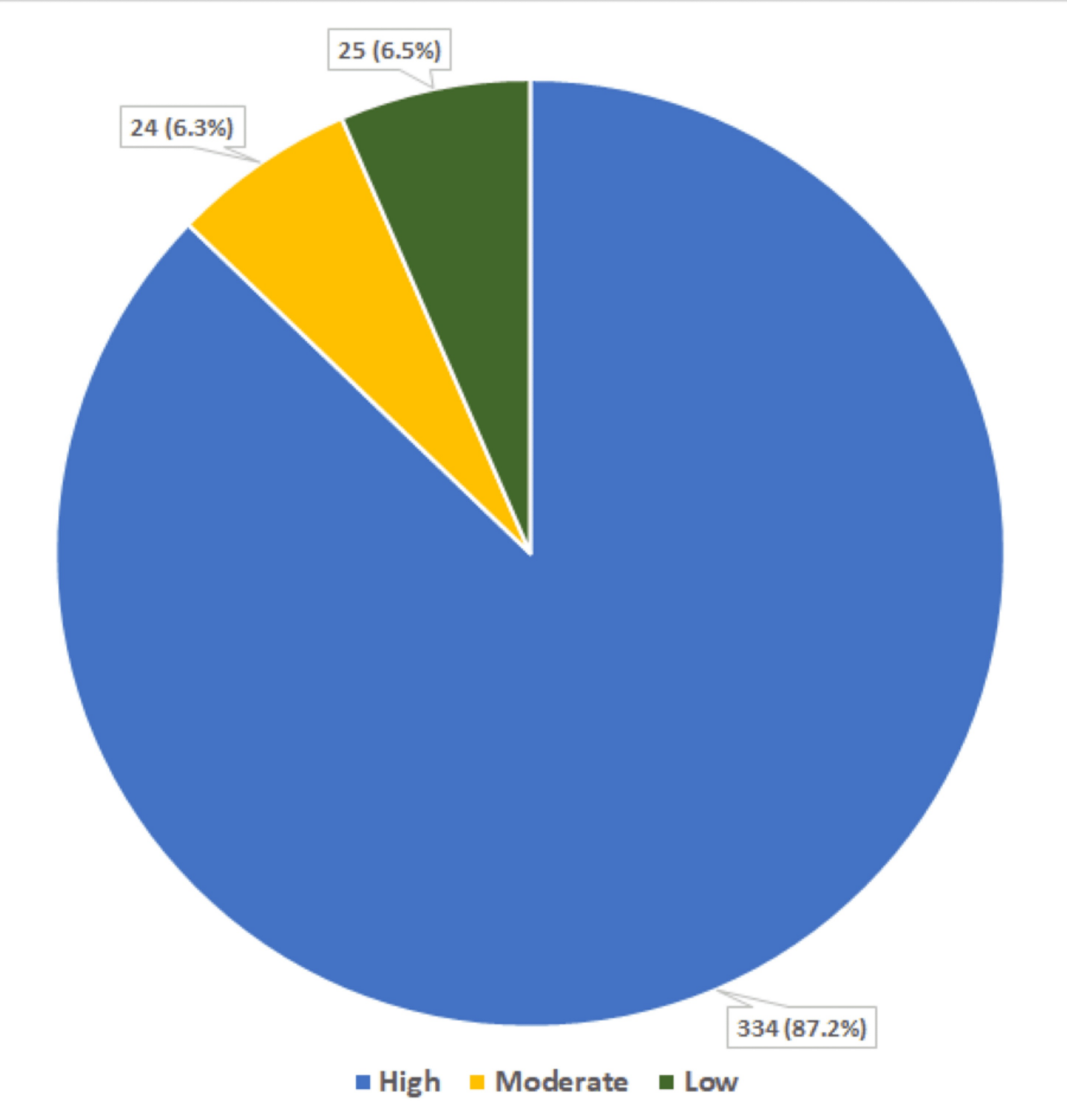
Awareness level among nursing officers

Figure 1 illustrates the awareness level regarding CNE among nursing officers. The majority of participants demonstrated a high level of awareness (334; 87.2%), indicating that most nursing officers had good knowledge and positive understanding regarding the importance of CNE programs. A smaller proportion of participants showed low awareness (25; 6.5%) and moderate awareness (24; 6.3%) toward CNE activities. The findings suggest that awareness regarding CNE among nursing officers was generally high.

## Discussion

The study findings demonstrated that the majority of nursing officers possessed high awareness and favorable attitudes toward CNE programs. Participants strongly agreed that continuing education is important for professional growth, updating clinical knowledge, and improving patient care quality. These findings support the observations of Pool et al. [1], who reported that nurses consider continuing professional development essential for maintaining professional competency and adapting to evolving healthcare demands. Similar findings were reported by Yu et al. [7], where nurses perceived continuing education as beneficial for improving confidence, clinical performance, and evidence-based practice.

The present study identified heavy workload, staff shortages, and insufficient time due to patient care responsibilities as the major barriers to participation in CNE activities. Fatigue after long duty hours and inconvenient timing of sessions were also commonly reported barriers. These findings are consistent with previous studies conducted in different healthcare settings. Chong et al. [3] found that workload burden, staffing limitations, and time constraints significantly reduced nurses’ participation in continuing education programs. Lim-Saco and Guino-o [5] also reported that insufficient administrative support and poor scheduling negatively influenced attendance in professional development activities.

Likewise, Wehabe et al. [9] highlighted workload pressure and lack of institutional support as major barriers affecting nurses’ engagement in continuing professional development. Similar evidence was reported by Aboshaiqah et al. [10], who observed that clinical workload and shortage of protected learning time reduced nurses’ participation in continuing education activities.

In the present study, clinically relevant topics, flexible scheduling, peer encouragement, supportive learning environments, and practical teaching methods emerged as important facilitators for participation in CNE programs. Nursing officers perceived interactive educational approaches such as simulations and case-based discussions positively. These findings are supported by Hakvoort et al. [6], who emphasized that organizational support, flexible educational opportunities, and accessibility of learning resources play a major role in motivating nurses toward continuing professional development. Bit-Lian et al. [11] similarly reported that nurses were more likely to participate in continuing education activities when educational content was directly related to clinical practice and delivered in a convenient format. Mlambo et al. [12] further highlighted that supportive workplace environments and lifelong learning opportunities improve nurses’ motivation, professional satisfaction, and competency.

The present study also demonstrated that awareness of CNE participation was not associated with demographic variables such as age, gender, or years of professional experience. This suggests that awareness and positive perception toward continuing education are consistently observed among nursing officers, irrespective of demographic differences. Similar findings were reported by Qalehsari et al. [2], who stated that nurses at various levels of experience recognize the importance of lifelong learning for maintaining clinical competency and professional development. Ross et al. [13] also observed that continuing professional development is widely accepted among nurses regardless of age and work experience.

The findings of the present study emphasize the importance of institutional support in improving participation in CNE programs. Measures such as adequate staffing, protected learning time, flexible scheduling, supervisor encouragement, and the availability of online learning opportunities may improve participation among nursing officers. Previous studies suggest that strengthening continuing professional education increases clinical competency, promotes patient safety, and improves the quality of healthcare services [14,15]. Continuous professional learning also helps clinical nurses adapt to evolving healthcare practices and supports the implementation of evidence-based nursing care [16]. Therefore, healthcare institutions should prioritize accessible, supportive, and practice-oriented CNE programs to encourage active participation as well as ongoing professional development among nursing officers.

Study limitation

The present study had certain limitations. First, the study was conducted at a single tertiary care center, which may limit the generalizability of the findings to other healthcare institutions or settings. Second, the use of a convenience sampling technique may have introduced selection bias, as participants were selected based on availability and willingness to participate. Additionally, the cross-sectional observational design of the study limited the ability to establish causal relationships between the identified barriers, facilitators, and participation in CNE programs.

Study implication

The findings of the present study have important implications for nursing practice, administration, and policy development. Workload, staff shortages, and time constraints were identified as major barriers, underscoring the need for adequate staffing support and protected learning time for nursing officers. Flexible scheduling, online learning opportunities, and interactive teaching strategies may improve participation in CNE programs. Nursing administrators should promote supportive learning environments through recognition and peer support. Strengthening accessible and practice-oriented CNE programs may enhance professional competency, evidence-based nursing practice, quality of patient care, and continuous professional development among nursing officers.

## Conclusions

The study concluded that nursing officers had good awareness and positive attitudes toward CNE programs. Heavy workload, staff shortage, and lack of time due to patient care responsibilities were identified as the major barriers affecting participation in CNE activities. Factors such as flexible scheduling, clinically relevant topics, peer encouragement, and supportive learning environments have been found to facilitate participation in CNE programs. The findings highlight the importance of organizational support and accessible educational opportunities in promoting continuous professional development among nursing officers. Strengthening CNE programs may contribute to improved clinical competency and quality patient care.

## Appendices

Self-structured questionnaire on barriers and facilitators influencing participation in Continuing Nursing Education (CNE) programs among nursing officers

Instructions

Please tick (✓) the most appropriate response. The information collected will be kept confidential and used only for research purposes.

Section A: Demographic characteristics

Section B: Awareness and attitude toward CNE (10 items)

Section C: Barriers to participation (10 items)

Section D: Facilitators for participation (10 items)

Section A: Demographic variables

1. Age (in years)

(a) Below 25  (b) 25-35  (c) 36-45  (d) Above 45

2. Gender

(a) Male  (b) Female  (c) Others

3. Years of professional experience

(a) Less than 1 year  (b) 1-5 years  (c) 6-10 years  (d) More than 10 years

4. Have you attended any CNE program in the past year?

(a) Yes  (b) No

Section B: Awareness and attitude toward Continuing Nursing Education (CNE)

Please tick (✓) the most appropriate response.

Scale: (a) Strongly agree  (b) Agree  (c) Neutral  (d) Disagree  (e) Strongly disagree

**Table 6:** Awareness and attitude toward Continuing Nursing Education (CNE)

Question	Strongly agree	Agree	Neutral	Disagree	Strongly disagree
I am aware of the CNE programs conducted in my hospital.					
CNE programs are important for professional development of nurses.					
Attending CNE programs improves the quality of patient care.					
The topics covered in CNE sessions are relevant to my clinical practice.					
The methods used in CNE sessions are effective for learning.					
Attending CNE programs motivates me to update my clinical knowledge.					
I feel confident applying knowledge gained from CNE sessions in my daily work.					
CNE participation should be mandatory for career advancement or promotion.					
The hospital management encourages participation in CNE sessions.					
I would recommend regular CNE attendance to my colleagues.					

Section C: Barriers to participation (these items focus only on difficulties and challenges)

Instruction: Rate how much each statement is a barrier for you.

Scale: (a) Strongly agree  (b) Agree  (c) Neutral  (d) Disagree  (e) Strongly disagree

**Table 7:** Barriers to participation (these items focus only on difficulties and challenges) CNE: Continuing Nursing Education

Question	Strongly agree	Agree	Neutral	Disagree	Strongly disagree
Heavy workload and staff shortage during duty hours					
Lack of time due to patient care responsibilities					
CNE sessions are scheduled at inconvenient times					
Inadequate information or short notice about sessions					
CNE topics are not related to my current work area					
Sessions are repetitive or not interesting					
Not received enough encouragement from supervisors					
Personal commitments sometimes prevent me from attending					
Fatigue or burnout after long shifts					
The venue/space or location of CNE is not convenient					

Section D: Facilitators for attending CNE (these items focus only on support and motivating factors)

Instruction: Rate how much each item would help you attend more CNEs.

Scale: (a) Strongly agree  (b) Agree  (c) Neutral  (d) Disagree  (e) Strongly disagree

**Table 8:** Facilitators for attending Continuing Nursing Education (CNE) (these items focus only on support and motivating factors)

Question	Strongly agree	Agree	Neutral	Disagree	Strongly disagree
Support from seniors inspires me to attend CNE					
Flexible or well-planned timing would increase my participation					
Duty adjustment or shift planning helps me participate					
Interactive and practical learning (case studies, simulations)					
Topics relevant to clinical practice and current updates					
Online or recorded sessions for those on duty/off shift					
Comfortable learning environment and facilities					
Peer encouragement and team participation					
Recognition or appreciation for attending CNEs					
Provision of handouts or learning materials					
